# Pediatric Holocord Epidural Abscess Treated with Apical Laminotomies with Catheter-directed Irrigation and Drainage

**DOI:** 10.7759/cureus.5733

**Published:** 2019-09-23

**Authors:** Elena Kurudza, James A Stadler

**Affiliations:** 1 Department of Neurological Surgery, University of Wisconsin, Madison School of Medicine and Public Health, Madison, USA

**Keywords:** spinal epidural abscess, pediatrics, spine surgery

## Abstract

Spinal epidural abscesses (SEA), while fortunately rare, carry significant risk to affected patients. Optimal treatment of these infections is poorly defined due to the heterogeneity of clinical and radiographic presentations. Urgent surgical evacuation of the infection is critical in cases with spinal cord compression or neurological compromise, though challenges may arise from competing surgical objectives, including the need for successful debridement of the infection, desire to minimize operative intervention, and risk of delayed iatrogenic instability. This is particularly concerning in young children with large multiregional collections. We present the first report case of pediatric holocord abscess treated with apical laminotomies and epidural catheterization for irrigation and drainage. This technique allowed successful treatment while avoiding extensive laminectomies and associated morbidities.

## Introduction

Spinal epidural abscess (SEA) is a rare but serious infection that requires prompt, definitive treatment to prevent a permanent neurologic deficit. Localized SEA is traditionally treated with urgent surgical decompression; medical management may be appropriate in select patients [1]. Treatment of extensive infections that are poorly responsive to medical management poses a particular challenge as the benefit of decompression and source control must be considered relative to the iatrogenic risk of a multilevel, and sometimes multiregional, laminectomy. Early experiences with lamina-sparing techniques in the adult population have been successful [2, 3]. However, this technique has not been described in the pediatric population, where there may be particular benefit from less invasive approaches with preservation of the growing anatomy, as extensive laminectomy in pediatric populations results in significant long-term morbidity [4].

We describe a case of an eight-month-old male with SEA and paraspinal abscess, with the extension of the infection from C2 to the sacrum, who failed medical management and was subsequently successfully treated with apical laminotomies with catheter-directed irrigation and drainage.

## Case presentation

History and exam

**Figure 1:**
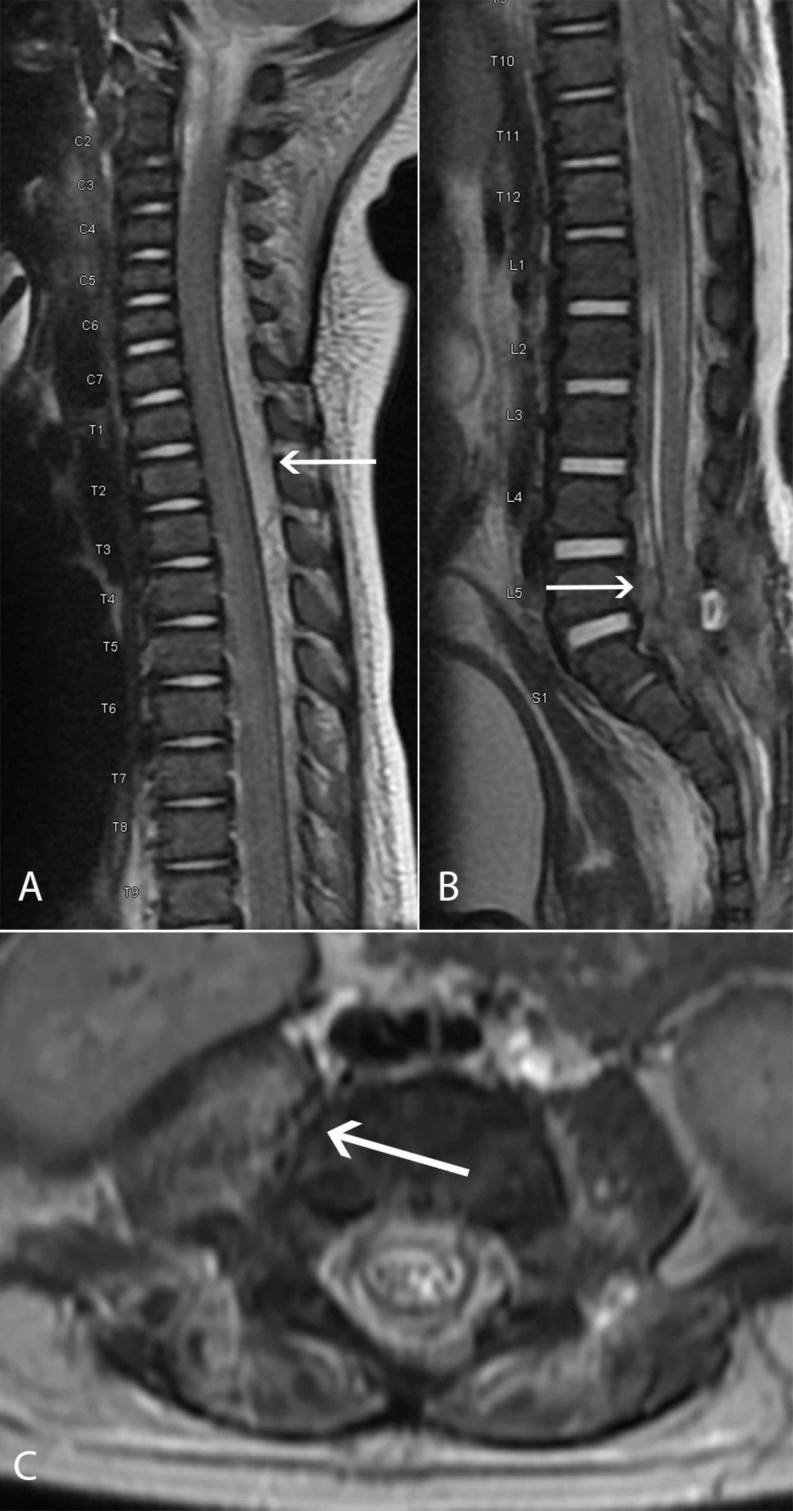
T2 MRI of the spine showing a significant epidural abscess extending from C2 to the sacrum, with cervicothoracic spinal cord compression (A), lumbosacral neural compression (B), and extension to the psoas/presacral musculature (C). Arrows demonstrate the epidural (A and B) and psoas abscesses (C).

A previously healthy eight-month-old male presented with fever, vomiting, irritability, and decreased oral intake. On exam, he was found to have signs of meningeal irritation. Of note, he did not demonstrate any focal neurologic deficit at the time of initial presentation. A lumbar puncture was attempted, though thick purulence was encountered prior to obtaining a cerebrospinal fluid sample; cultures demonstrated methicillin-resistant Staphylococcus aureus (MRSA). He was admitted to the pediatric intensive care unit and antibiotic therapy was initiated. Two days later, however, he was noted to be acutely paraplegic and diffusely hypotonic, prompting neurosurgical consultation. MRI of the spine demonstrated an extensive epidural fluid collection extending from upper cervical spine to lumbar spine and focal epidural thickening at the L5-S1 level that communicated with a peripherally enhancing 2.4 cm abscess in the paraspinal and psoas musculature. There was upper thoracic spinal cord compression and T2 cord signal abnormality noted in the cervical and thoracic spine (Figure 1).

**Figure 2:**
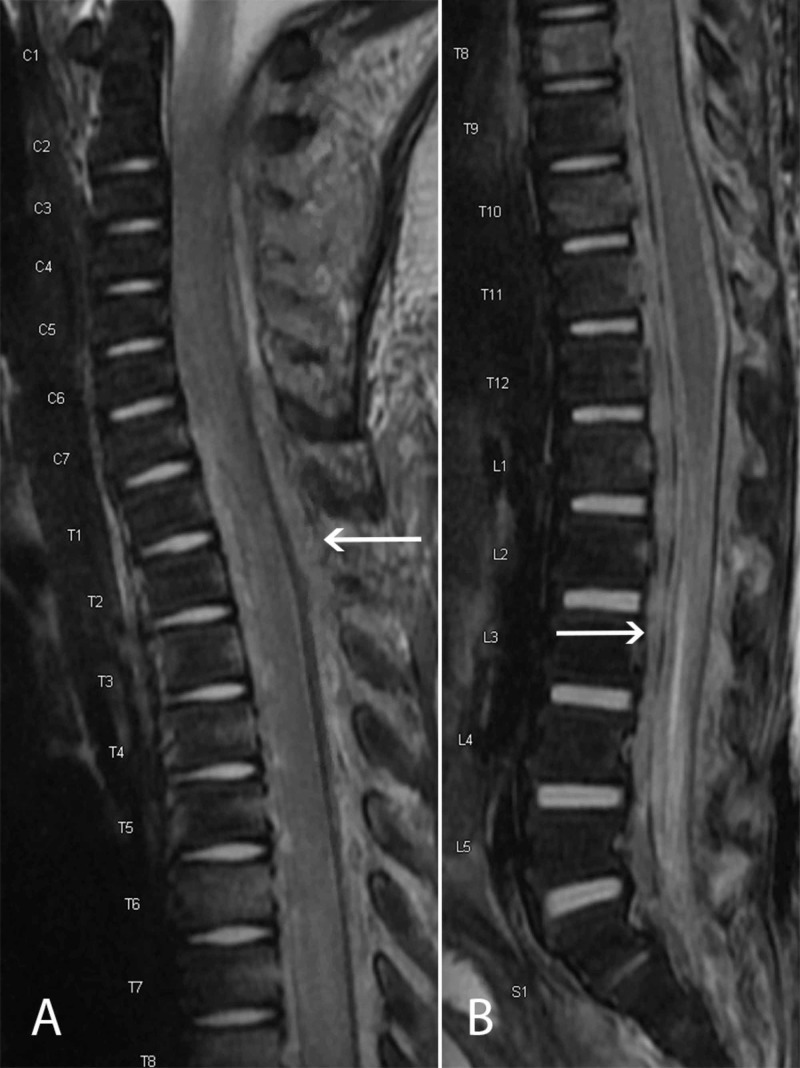
Interval T2 MRI following initial decompression with recurrent/residual extensive spinal epidural abscess in the cervicothoracic (A) and lumbosacral (B) regions

The patient was taken to surgery for an emergent T3 laminotomy for decompression. He tolerated this procedure well. He initially did well postoperatively, with the return of normal muscle tone and motor function, and appropriate pathogen-directed antibiotic therapy was continued. Repeat MRI of the spine was obtained three days later due to fluctuating neurologic exam, persistent fevers, and redemonstration of MRSA bacteremia after initial clearance (Figures 2). This demonstrated worsening of the extraspinal abscess and recurrence of the epidural fluid collection. Percutaneous drains were placed by the interventional radiology team for attempted source control of the presacral and psoas abscesses. However, given limited response to medical management and continued intermittent myelopathy on examination, the patient returned to surgery on hospital day 12 for apical laminotomies with catheter-directed irrigation and drainage. 

Operation

**Figure 3:**
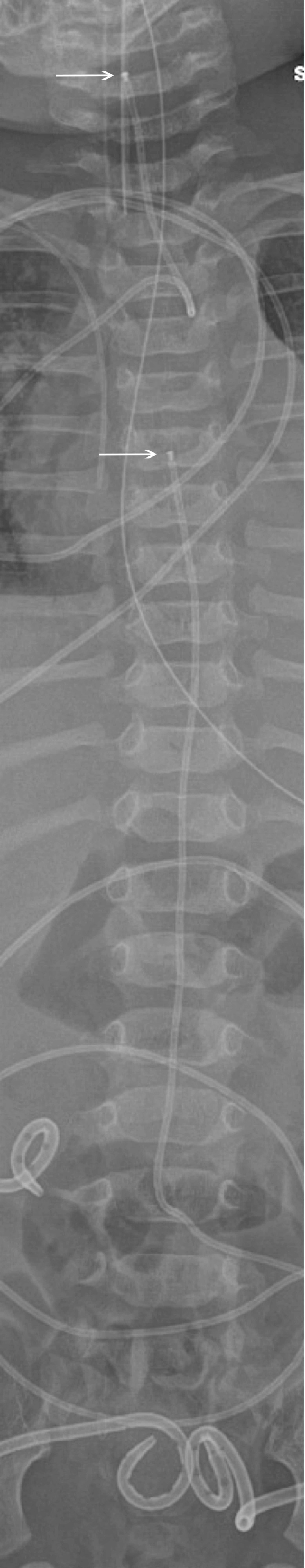
Postoperative anteroposterior spinal radiograph demonstrating dual epidural catheter placement via T3 and L4 laminotomies, with the respective catheter tips demarcated by arrows

Laminotomies were made at T3 and L4 to expose the epidural space, with the utilization of the prior incision and laminotomy at T3. The local epidural spaces were debrided and irrigated in a standard fashion. Given the diffuse extent of the epidural abscess, extensive multiregional laminotomies/laminectomies were felt to carry significant risk for iatrogenic deformity, and we, therefore, attempted irrigation and debridement of the epidural space with a lumbar drainage catheter. This was introduced into the epidural space via the respective laminotomies and guided cranially and caudally from each location, with systematic irrigation of purulent fluid and phlegmon from each region. Normal saline was used for initial debridement, with irrigation continued until clear effluent from each laminotomy was noted to ensure appropriate communication; we subsequently irrigated with bacitracin- and vancomycin-containing solutions as well. Attention to the manual irrigation pressure and close monitoring of relative output minimized the risk of iatrogenic spinal cord compression. An epidural catheter was directed cranially from each laminotomy for continued postoperative drainage (Figure 3).

Postoperative course

**Figure 4:**
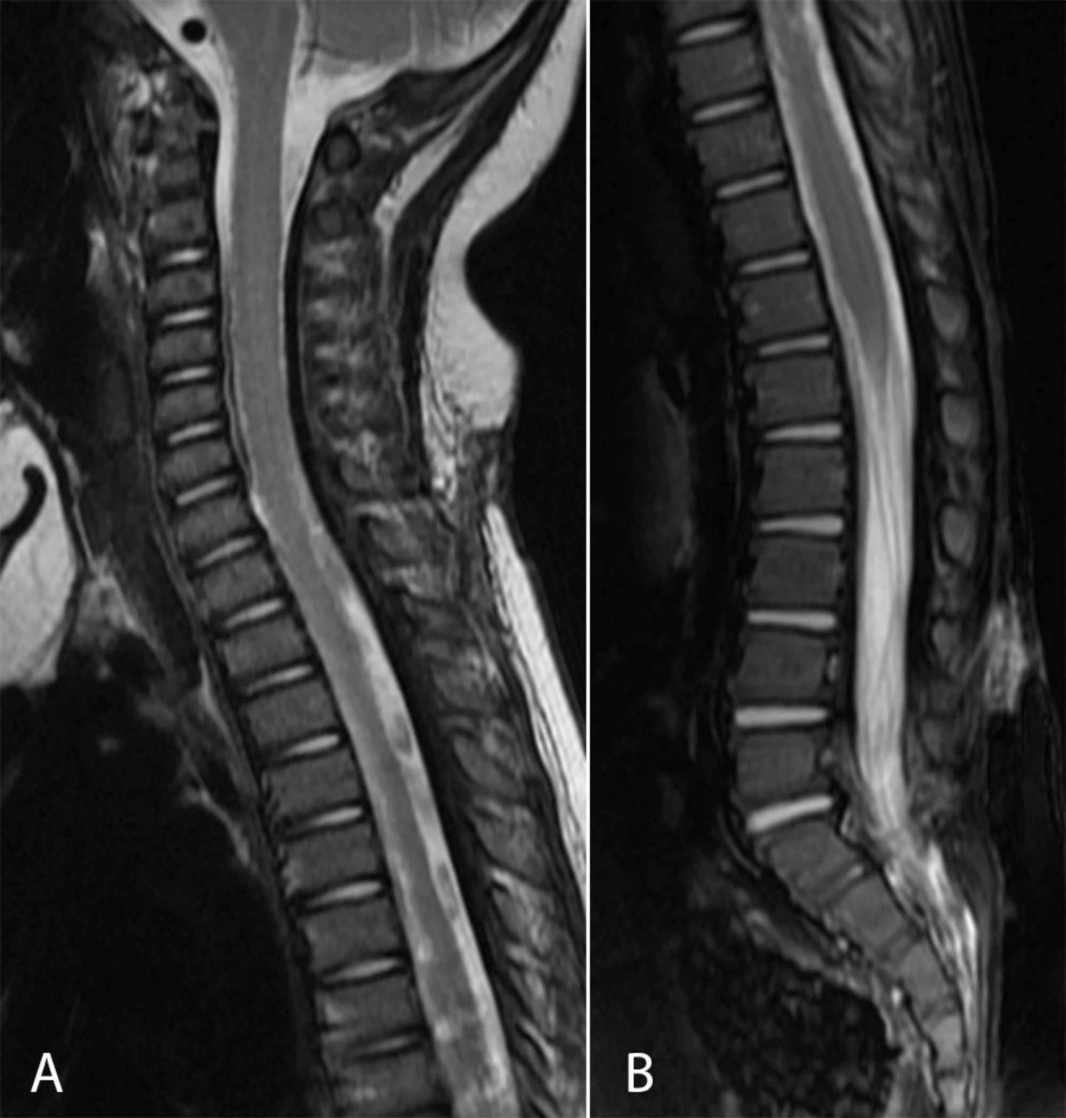
Postoperative T2 MRI of the cervicothoracic (A) and lumbosacral (B) regions with resolution of the previously noted epidural abscess

The patient did well postoperatively with normalization of his neurological exam. On postoperative day 2 both epidural drains were removed after output was decreased. The patient exhibited resolution of his fevers and leukocytosis, and he had significant improvement in inflammatory markers. By postoperative day 4 he was transferred to general care, with subsequent discharge home on postoperative day 14. He continued intravenous antibiotics for a total four-week course. MRI demonstrated resolution of the epidural abscess (Figure 4), and he continues to do well clinically with appropriate development at least six months following these treatments. 

## Discussion

Optimal treatment of SEA lacks consensus, being confounded by significant heterogeneity in clinical and radiographic presentations. A systematic review published in 2014 by Arko et al. reported no significant difference in outcomes between operative and nonoperative management in patients presenting without neurologic deficit or spinal instability [1]. However, Curry et al. observed improved outcomes with urgent surgical decompression over conservative, nonoperative management [5]. In their single-institution retrospective analysis, 49% of their patients who were initially managed nonoperatively decompensated during their hospitalization and required urgent surgical decompression, thus strengthening an argument for early surgical management.

Management decisions become further complicated in the case of extensive SEA. The available literature is limited due to the relative rarity of this presentation, and extended decompression carries greater operative and delayed iatrogenic risks. Management options include skip laminectomies, laminoplasties, percutaneous drainage, and medical management [6-8]. More recently, apical laminotomies with irrigation and drainage have been described in a small number of adult patients [2, 3].

Consideration of delayed complications is particularly important in the pediatric population given skeletal immaturity and greater remaining life expectancy. SEA in children is even rarer than in the adult population, and holocord involvement makes up a small minority of these cases [7, 9]. Laminectomy/laminoplasty involving greater than four levels is known to be associated with increased risk of spinal deformity that necessitates surgical fusion in a pediatric population [4]. Thus, lamina-sparing approaches to pediatric SEA may be particularly important in the prevention of significant iatrogenic morbidity in this population.

Ideally, treatment of extensive SEA in children results in resolution of the infection and neural element decompression while minimizing surgical trauma to the growing spine. Here we present a case of a young child successfully treated with apical laminotomies and catheter-directed irrigation and debridement. This technique achieved traditional surgical objectives while avoiding more extensive spinal intervention.

## Conclusions

We describe the successful resolution of an extensive SEA with apical laminotomies and catheter-directed irrigation and drainage in a pediatric patient. To our knowledge, this is the first reported application of this method has been described in the pediatric population. Based on these findings and the known benefits of avoiding extended multilevel decompression, we encourage consideration of apical laminotomies with catheter-directed irrigation and drainage in pediatric patients with extensive SEA.
